# Evaluating Exercise Progression in an Australian Cardiac Rehabilitation Program: Should Cardiac Intervention, Age, or Physical Capacity Be Considered?

**DOI:** 10.3390/ijerph18115826

**Published:** 2021-05-28

**Authors:** Kym Joanne Price, Brett Ashley Gordon, Stephen Richard Bird, Amanda Clare Benson

**Affiliations:** 1 Discipline of Exercise Sciences, School of Health and Biomedical Sciences, RMIT University, Melbourne, VIC 3083, Australia; stephen.bird@rmit.edu.au; 2 Holsworth Research Initiative, La Trobe Rural Health School, La Trobe University, Bendigo, VIC 3550, Australia; b.gordon@latrobe.edu.au; 3 Department of Health and Biostatistics, Sport Innovation Research Group, Swinburne University of Technology, Melbourne, VIC 3122, Australia; abenson@swin.edu.au

**Keywords:** exercise prescription, exercise capacity, cardiovascular disease, coronary artery bypass surgery

## Abstract

Progression of prescribed exercise is important to facilitate attainment of optimal physical capacity during cardiac rehabilitation. However, it is not clear how often exercise is progressed or to what extent. This study evaluated whether exercise progression during clinical cardiac rehabilitation was different between cardiovascular treatment, age, or initial physical capacity. The prescribed exercise of sixty patients who completed 12 sessions of outpatient cardiac rehabilitation at a major Australian metropolitan hospital was evaluated. The prescribed aerobic exercise dose was progressed using intensity rather than duration, while repetitions and weight lifted were utilised to progress resistance training dose. Cardiovascular treatment or age did not influence exercise progression, while initial physical capacity and strength did. Aerobic exercise intensity relative to initial physical capacity was progressed from the first session to the last session for those with high (from mean (95%CI) 44.6% (42.2–47.0) to 68.3% (63.5–73.1); *p* < 0.001) and moderate physical capacity at admission (from 53.0% (50.7–55.3) to 76.3% (71.2–81.4); *p* < 0.001), but not in those with low physical capacity (from 67.3% (63.7–70.9) to 85.0% (73.7–96.2); *p* = 0.336). The initial prescription for those with low physical capacity was proportionately higher than for those with high capacity (*p* < 0.001). Exercise testing should be recommended in guidelines to facilitate appropriate exercise prescription and progression.

## 1. Introduction

Cardiovascular disease (CVD) is a leading cause of morbidity worldwide, with major economic and personal implications due to increased health care costs and the loss of patient and carer productivity [1]. Cardiac rehabilitation is a cost-effective intervention that reduces cardiovascular morbidity, repeat hospital admissions and mortality, and facilitates individuals with CVD to recover and attain their optimal functional capacity via improvements in aerobic fitness and muscular strength [2].

Cardiac rehabilitation programs are typically implemented across 3–12 weeks in Australia and up to 24 weeks internationally [3]. Often, functional assessments are not re-evaluated at the end of the program and individuals are discharged after completing the set number of sessions rather than after achieving any objectively measured outcome [4]. Therefore, it remains uncertain if patients have attained their full potential before ‘graduating’ from the cardiac rehabilitation program. Potential consequences of this include patients functioning at a sub-optimal level in their activities of daily living and personal interests, which can impact on their quality of life physically as well as increase the risk of social isolation, anxiety, and depression, and subsequent cardiac events [1].

Following medical intervention for cardiovascular disease, individuals typically have a lower physical capacity (approximately five to six METs) [5,6] compared with healthy age-matched individuals (approximately nine METs) [7,8]. Greater impairments in function are observed in individuals who have received surgical intervention as opposed to non-surgical management [5,9]. Therefore, completion of activities of daily living can require a high percentage of a cardiac patient’s physical capacity and a large amount of exertion [5]. Indeed the development or progression of physical limitations and disability often occurs following hospitalisation or restricted activity, as is common following an acute cardiac event [9]. This is of concern and highlights the importance of cardiac rehabilitation programs enabling these patients to achieve their full physical potential. This goal can only be reached if the prescribed exercise program is of an appropriate type, intensity, frequency, and duration, and applies the principle of progression as the patients’ physical capacities improve during the program. This may be achieved via structured exercise sessions in cardiac rehabilitation, but they should be individually tailored to allow for the safe progression of exercise dose to provide gradual overload as the body adapts to the stress of training [10].

It has recently been demonstrated that the initial exercise prescription in an Australian cardiac rehabilitation program considers patient symptoms more than measured physical capacity [11]. Additionally, it is not clear how exercise prescription is progressed over the duration of cardiac rehabilitation. The Australian guidelines for exercise prescription in cardiac rehabilitation encourage review of individual exercise prescription on a minimum of three occasions throughout the program, but do not provide any information regarding progressing the prescription [12]. This is similar for cardiac rehabilitation guidelines prepared by other national organisations [3]. Exercise progression (progressive overload) is necessary to ensure continued adaptations to the stress of training, and achievement of further gains in physical capacity and cardiovascular risk reduction. In comparison, a lack of progression is likely to result in a plateau with gains lower than what might be possible.

Therefore, the aims of this study were to investigate the frequency and volume of exercise progression over the course of an outpatient cardiac rehabilitation program in Australia, and whether the exercise progression is different between cardiovascular intervention received, age or initial physical capacity category.

## 2. Materials and Methods

Patients who attended outpatient cardiac rehabilitation at a major metropolitan hospital in Australia following medical intervention for an acute coronary event were invited to participate in the study. Patients were assessed against the exclusion criteria (presence of unstable cardiac disease including unstable angina, severe or critical aortic stenosis, or aortic aneurysm, co-morbidities that increase the risk of complications with exercise including severe un-medicated pulmonary artery hypertension, an inability to understand written and/or spoken English, or significant cognitive impairments that limited patient understanding or ability to follow instructions) by the program’s cardiac nurse, and provided written informed consent prior to their participation. The study was approved by the Austin Health Human Research Ethics Committee (reference number: 05146) and RMIT University Human Research Ethics Committee (reference number: H2013/005146).

Demographic data (sex, age, and cardiovascular intervention) were collected, with cardiovascular intervention categorised into surgical (coronary artery bypass graft or valve surgery), and non-surgical (percutaneous coronary intervention or medical management) interventions. Patients were categorised as younger (<65 years) or older (≥65 years) to align with the age categories upon which the classification of exercise intensity is based in the Australian cardiac rehabilitation guidelines [12].

Height and body mass were measured using a wall-mounted stadiometer (Surgical and Medical Supplies Pty. Ltd., Adelaide, Australia) and digital scales (Tanita HD-316, Arlington Heights, IL, USA), respectively, according to standardised procedures [13]. Body mass index (BMI) was calculated as body mass (kg)/height (m)^2^ [10].

Prior to commencement and upon completion of the cardiac rehabilitation program, aerobic physical capacity was assessed using the incremental shuttle walk test (ISWT) [14]. This is a standardised symptom-limited test that is frequently used in cardiac rehabilitation programs to determine physical capacity [6,15]. On both occasions, the test was conducted according to the protocol described by Singh, et al. [14] by the same single assessor not associated with delivery of cardiac rehabilitation, and was terminated when patients were unable to maintain the required walking speed, or if the patient self-selected to stop. Total distance walked was converted to metabolic equivalents (METs) using a published regression equation [15]. Muscle strength was assessed using a digital hand-grip dynamometer (Jamar Plus+, Patterson Medical, Warrenville, IL, USA).

The cardiac rehabilitation program consisted of 12 sessions with patients typically attending the program twice weekly over six weeks. Extension of the program was permitted where patients had been absent from a session due to illness, vacation, or program closure. All exercise sessions were held indoors in a hospital-based clinical gymnasium, and were prescribed and supervised by a combination of physiotherapy, exercise physiology, and nursing staff.

Cardiac rehabilitation exercise sessions were one hour, and included up to 30 min of aerobic training, comprising of walking and stationary cycling. For walking, use of a treadmill was the preferred option, although for older patients and those unfamiliar with treadmill use, walking a 50 m circuit over flat ground was available. Clinical judgement was used to guide initial exercise prescription at low- to moderate-intensity in accordance with the national guidelines for cardiac rehabilitation [12]. Resistance training included three different dumbbell exercises (shoulder press, upright row, sit-to-stand with bicep curl) and was prescribed using light weights (1–5 kg). Borg’s rating of perceived exertion (RPE) CR10 scale was used to monitor the intensity of exercise training [16]. Exercise intensity was progressed throughout the cardiac rehabilitation program using clinical judgement based on RPE and observations made by supervisors during the exercise sessions.

Exercise training data were recorded by cardiac rehabilitation program staff. Speed, incline, and duration were recorded for treadmill walking, along with distance and duration for overground walking. Cycling intensity in Watts and duration were recorded for cycling where possible; however, several brands of cycles were utilised in the program and some of these were not able to set a specific work rate in Watts. Therefore, there are differences in the number of patients contributing to analyses for cycling intensity/dose and duration. The absolute intensity for each aerobic exercise modality was calculated for each exercise session using the American College of Sports Medicine metabolic equations for walking and leg cycling [10]. Walking and cycling absolute intensity were multiplied by the duration of the activity to determine the exercise dose in MET·min, with walking and cycling dose summed to obtain the total aerobic exercise dose. Relative aerobic training intensity was calculated for each session relative to the individual’s initial physical capacity estimated from ISWT. Relative aerobic training intensity was also calculated for the final session relative to final physical capacity. Weight lifted, sets, and repetitions were recorded for resistance training. Resistance training load (kg) was calculated as weight lifted multiplied by sets and repetitions [17].

Patients were included for analysis if they had exercise training data recorded for at least 75% of the available exercise sessions. Where recorded training information was incomplete, data were carried over from the previous session. This conservatively estimated no progression to the exercise prescription.

Statistical analysis was undertaken using IBM SPSS Statistics 25 (IBM Corp., Armonk, NY, USA). Data are presented as mean ±SD or mean (95% confidence interval). Normal distribution was confirmed using the Shapiro-Wilk test and visual examination of histograms. To enable comparisons of exercise prescription received between different baseline physical capacity levels, patients were categorised based on their initial physical capacity (<5 METs, 5–7 METs, >7 METs) according to the risk categories described in the American Association of Cardiovascular and Pulmonary Rehabilitation guidelines for cardiac rehabilitation [18], as well as distributed into tertiles based on initial grip strength.

Two-way repeated measures ANOVAs were conducted to test for differences in prescribed exercise intensity, duration and dose between initial physical capacity categories, cardiovascular interventions, and age over the duration of cardiac rehabilitation. Where assumptions of sphericity were violated, the Greenhouse-Geisser correction was applied. Bonferroni corrections were applied to adjust for multiple comparisons. When significant time by group interactions were identified, one-way ANOVAs were conducted to identify the differences in exercise progression between physical capacity categories, cardiovascular interventions, and age. To assess whether relative aerobic exercise intensity was different at the end of the program compared to the start of the program paired *t*-tests were conducted. A value of *p* < 0.05 was considered statistically significant.

## 3. Results

### 3.1. Participant Demographics

Eighty-two patients who attended the cardiac rehabilitation program between July 2015 and August 2016 commenced the study. Sixty patients completed a minimum of nine exercise sessions (75% of the scheduled 12 sessions) and were included in the analyses. Baseline demographic information for included participants is shown in Table 1.

### 3.2. Aerobic Exercise Training Progression

**Figure 1:**
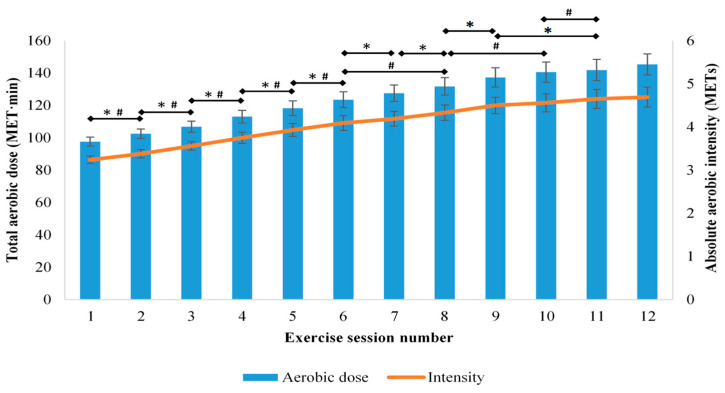
Aerobic exercise training prescription during 12 sessions of cardiac rehabilitation. Data presented as mean (95% CI). * Significant progression in total exercise dose, ^#^ significant progression in absolute aerobic intensity. METs—metabolic equivalents.

The mean (95% CI) total aerobic exercise dose increased from the first session (98 (95 to 100) MET·min) to the final session (145 (139 to 152) MET·min). This progression appeared to be continuous throughout the program, as there were increases in each of the first nine sessions compared to the previous session, and in session 11 from session 9 (F_(2 40, 129.43)_ = 202.49, *p* < 0.001; Figure 1). Additionally, there was a significant interaction between time and age for total aerobic dose (F_(2.61, 138.50)_ = 5.15, *p* < 0.001), which indicated that there was less frequent progression of the exercise prescription for older patients than younger patients. Progression of aerobic exercise dose over the course of the program was also influenced by initial physical capacity category (F_(5.25, 136.48)_ = 4.58, *p* = 0.001), with progression identified in those with moderate and high initial physical capacity, but not low physical capacity. There was no interaction between time and cardiovascular intervention (surgical or non-surgical) for total aerobic exercise dose (F_(2.38, 126.10)_ = 0.28, *p* = 0.797).

The mean (95% CI) walking dose increased from the first session (45 (43 to 48) MET·min) to the final session (73 (67 to 78) MET·min) with frequent progression from session to session, up to session 10 (F_(3.00, 171.08)_ = 80.07, *p* < 0.001). The mean (95% CI) cycling dose also increased from the first session (51 (49 to 52) MET·min) to the final session (73 (70 to 76) MET·min), with frequent progression from session to session, up to the final session (F_(3.02, 153.78)_ = 180.09, *p* < 0.001). Neither walking (F_(2.47, 133.36)_ = 0.922, *p* = 0.417) nor cycling duration (F_(1.35, 71.27)_ = 2.11, *p* = 0.144) changed throughout the program, which indicated that the observed progression was due to increased exercise intensity.

**Figure 2:**
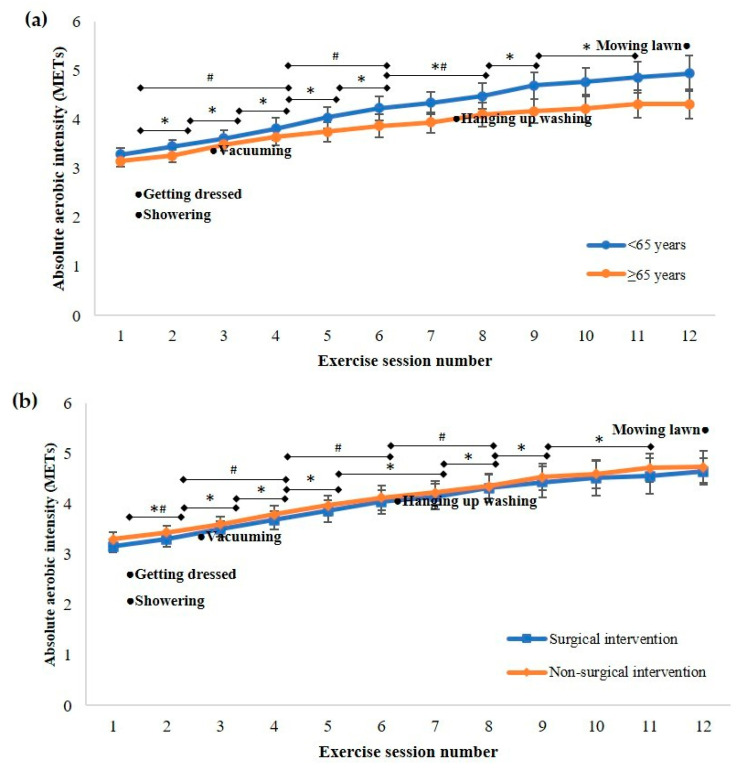

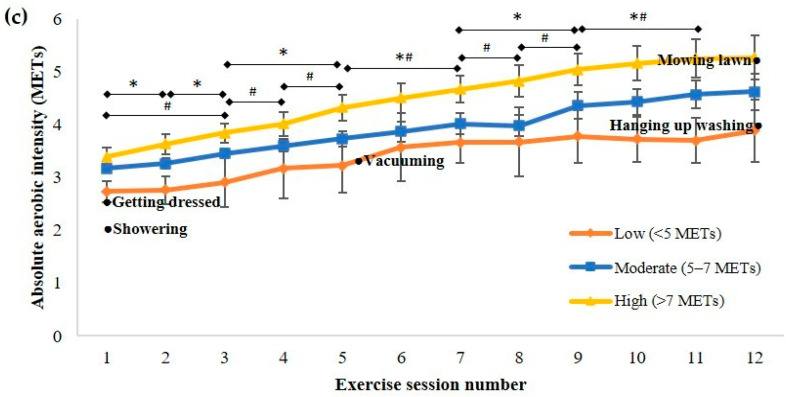
Comparison of the absolute aerobic intensity in metabolic equivalents (METs) prescribed during cardiac rehabilitation sessions with the requirements of activities of daily living, for patients categorised by (**a**) age; (**b**) cardiovascular intervention; and (**c**) initial physical capacity. Data presented as mean (95% CI). * Significant progression in absolute aerobic intensity for (**a**) <65 years; (**b**) non-surgical intervention; and (**c**) high physical capacity. ^#^ Significant progression in absolute aerobic intensity for (**a**) ≥65 years; (**b**) surgical intervention; and (**c**) moderate physical capacity.

The mean (95% CI) absolute aerobic intensity increased from the first session (3.2 (3.1 to 3.3) METs) to the final session (4.7 (4.5 to 4.9) METs), with increases occurring in each of the first six sessions compared to the previous session, in session 8 compared to session 6, in session 10 compared to session 8, and in session 11 compared to session 10 (F_(2.51, 148.13)_ = 145.71, *p* < 0.001; Figure 1). There was a significant interaction between time and age for absolute aerobic intensity (F_(2.69, 156.12)_ = 4.61, *p* = 0.006), with more frequent progression of intensity occurring for younger patients. The mean absolute intensity of aerobic exercise prescribed to patients aged less than 65 years progressed over the course of the program from 3.3 (3.2–3.4) METs (which is classified as light- intensity (2.0–3.9 METs) according to the Australian guidelines for cardiac rehabilitation [12] for this age group) [12] to 4.9 (4.6–5.2) METs, which is defined as a moderate-intensity (4.0–5.9 METs) for this <65 years age group. However older patients were initially prescribed an exercise intensity of 3.2 (3.0–3.3) METs, which is defined as moderate-intensity aerobic exercise (3.2–4.7 METs) for those >65 years and at the end of the program were being prescribed an intensity of 4.3 (4.0–4.7) METs, which remained in this moderate intensity zone (Figure 2a). There was no interaction between time and cardiovascular intervention for absolute aerobic intensity (F_(2.50, 145.02)_ = 0.175, *p* = 0.883; Figure 2b).There was also a significant interaction between time and initial physical capacity category for absolute aerobic intensity (F_(5.83, 166.23)_ = 6.50, *p* < 0.001), with progression of intensity identified in patients in the moderate and high initial physical capacity categories, but not in the lowest initial physical capacity category. The absolute aerobic intensity prescribed to patients with an initial physical capacity greater than 5 METs progressed from light to moderate over the course of their cardiac rehabilitation program (Figure 2c).

The mean (95% CI) absolute walking intensity increased from the first session (3.1 (3.0 to 3.2) METs) to the final session (4.7 (4.4 to 5.0) METs), with no increase in intensity after session 9 (F_(2.57, 149.12)_ = 91.78, *p* < 0.001). The mean (95% CI) absolute cycling intensity increased from the first session (3.4 (3.3 to 3.5) METs) to the final session (4.9 (4.7 to 5.1) METs), with increases in each of the first ten sessions compared to the previous session, and in session 12 compared to session 10 (F_(2.80, 145.54)_ = 192.63, *p* < 0.001).

**Figure 3:**
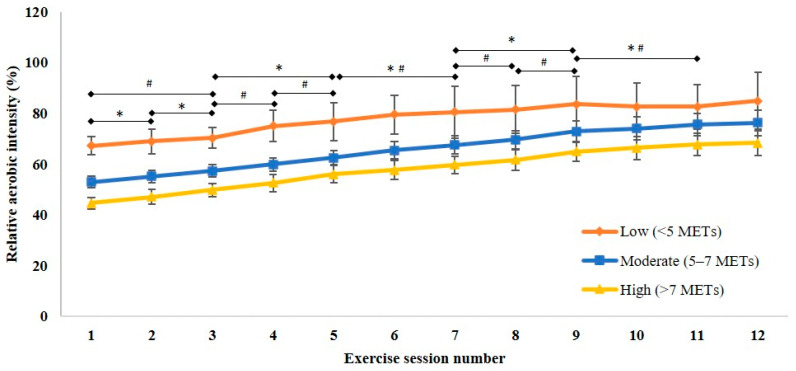
Relative (to initial physical capacity) aerobic exercise intensity prescribed to patients with different initial physical capacity during 12 sessions of cardiac rehabilitation. Data presented as mean (95% CI). * Significant progression in relative aerobic intensity for high initial physical capacity, ^#^ significant progression in relative aerobic capacity for high initial physical capacity. METs—metabolic equivalents.

For relative (to initial physical capacity) aerobic intensity, there was an increase in the mean (95% CI) from the first session (52% (50 to 55)) to the final session (75% (71 to 78)), with increases occurring in each of the first nine sessions compared to the previous session, and in session 11 compared to session 9 (F_(2.23, 129.07)_ = 181.77, *p* < 0.001; Figure 3). For relative (to initial physical capacity) aerobic intensity, there was no interaction between time and age (F_(2.29, 130.65)_ = 2.19, *p* = 0.109), between time and cardiovascular intervention (F_(2.27, 129.58)_ = 1.81, *p* = 0.162), or between time and initial physical capacity category (F_(4.44, 124.35)_ = 1.28, *p* = 0.281). Relative (to initial physical capacity) aerobic intensity was progressed from the first session to the last session for those with high (from mean (95%CI) 44.6% (42.2–47.0) to 68.3% (63.5–73.1); *p* < 0.001) and moderate initial physical capacity (from 53.0% (50.7–55.3) to 76.3% (71.2–81.4); *p* < 0.001), but not in those with low initial physical capacity (from 67.3% (63.7–70.9) to 85.0% (73.7–96.2); *p* = 0.336). The relative aerobic intensity prescribed in the first session for those with low initial physical capacity was proportionately higher than for those with high initial physical capacity (*p* < 0.001). When aerobic intensity at the end of the program was expressed relative to final physical capacity (mean (95% CI) 64% (61 to 67)), progression from the initial relative intensity (relative to initial physical capacity) was still evident (t_(55)_ = −9.28, *p* < 0.001).

### 3.3. Resistance Training Progression

**Figure 4:**
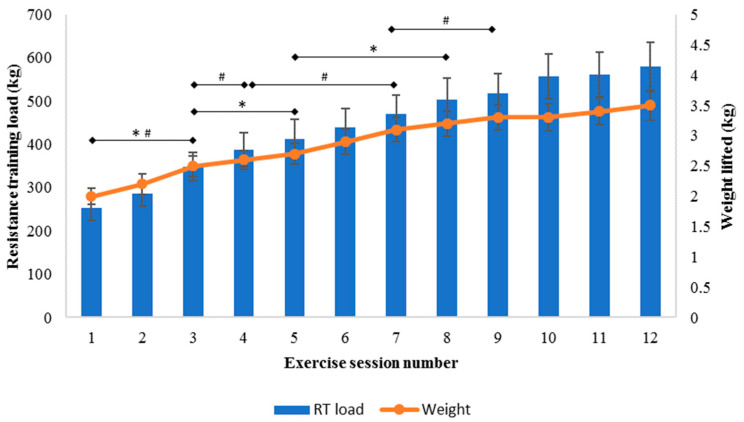
Resistance training (RT) prescription during 12 sessions of cardiac rehabilitation. Data presented as mean (95% CI). * Significant progression in resistance training load, ^#^ significant progression in weight lifted during resistance training.

The mean (95% CI) resistance training load (where resistance training load = weight lifted multiplied by sets and repetitions) increased from the first session (254 (225 to 283) kg) to the final session (579 (522 to 637) kg), with increases in session 3 compared to session 1, in session 5 compared to session 3, and in session 8 compared to session 5 (F_(4.40, 218.19)_ = 56.45, *p* < 0.001; Figure 4). Resistance training load remained similar after session 8. There was a significant interaction between time and initial grip strength tertile for resistance training load (F_(9.26, 217.52)_ = 2.19, *p* = 0.023). Resistance training load increased twice throughout the program for those in the highest and middle tertiles, but only once for those in the lowest tertile for initial grip strength. For resistance training load, there was no interaction between time and age (F_(4.34, 212.63)_ = 0.45, *p* = 0.787), or between time and cardiovascular intervention (F_(4.30, 210.66)_ = 1.37, *p* = 0.244).

**Figure 5:**
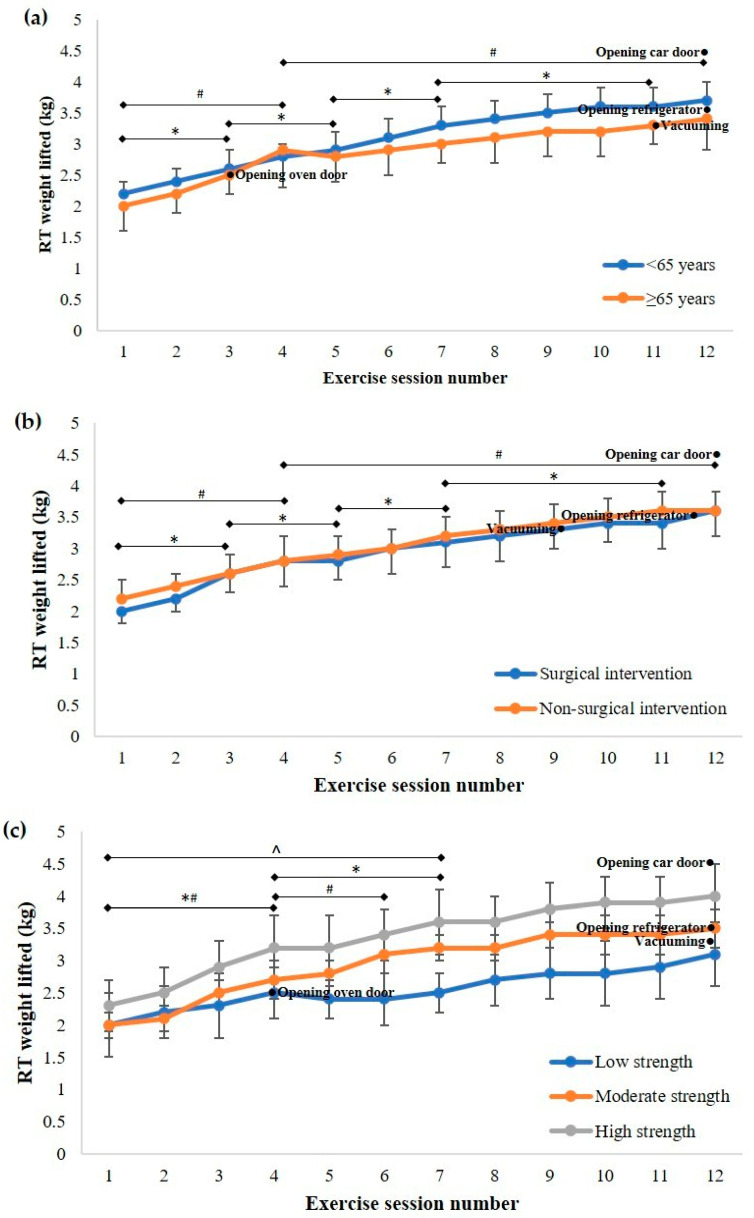
Comparison of resistance training (RT) weight lifted during cardiac rehabilitation with work required for activities of daily living, with patients categorised by (**a**) age; (**b**) cardiovascular intervention; and (**c**) initial grip strength tertile. Data presented as mean (95% CI). * Significant progression in weight lifted for (**a**) <65 years; (**b**) non-surgical intervention; and (**c**) high physical capacity. ^#^ Significant progression in weight lifted for (**a**) ≥65 years; (**b**) surgical intervention; and (**c**) moderate physical capacity. ^^^ Significant progression in weight lifted for (**c**) low physical capacity.

The mean (95% CI) weight lifted during resistance training increased from the first session (2.0 (1.9 to 2.1) kg) to the final session (3.5 (3.3 to 3.8) kg), but did not change after session 9 (F_(3.17, 148.76)_ = 71.54, *p* < 0.001; Figure 4). For resistance training weight lifted, there was no interaction between time and age (F_(3.16, 145.19)_ = 0.51, *p* = 0.683), time and cardiovascular intervention (F_(3.15, 144.78)_ = 0.26, *p* = 0.862), or time and initial grip strength (F_(6.60, 145.10)_ = 1.87, *p* = 0.083). Weight lifted during resistance training compared to activities of daily living is presented in Figure 5.

The mean (95% CI) number of resistance training repetitions increased from the first session (9.9 (9.6 to 10.3) repetitions) to the final session (12.1 (11.5 to 12.8) repetitions), with the only significant increase in the number of repetitions occurring in session 5 compared to session 1 (F_(4.78, 239.16)_ = 9.39, *p* < 0.001). However, there was no significant difference in the number of resistance training sets prescribed from the start to the end of the program (F_(4.13, 202.56)_ = 2.15, *p* = 0.073). There was a significant interaction between time and cardiovascular intervention (F_(4.82, 236.06)_ = 2.31, *p* = 0.048) for the number of resistance training repetitions, with progression identified in the non-surgical patients but not in those who had received surgical intervention. For resistance training repetitions, there was no interaction between time and age (F_(4.90, 239.93)_ = 1.38, *p* = 0.235), or time and initial grip strength (F_(9.38, 220.51)_ = 0.52, *p* = 0.865).

## 4. Discussion

In summary, there were inconsistencies in the relative exercise intensity and dose prescription and progression for different patients. The implications of this are that some patients may not be experiencing an optimal training stimulus throughout the program and upon completion may not have attained their full potential. The patient’s full potential might be realised through employing a complete individual tailoring of exercise prescription that fully considers initial and changes to physical capacity in addition to patient symptoms and motivation when prescribing exercise dose, and/or a longer program duration (more sessions) that would enable continued progression and adaptations.

The final mean aerobic exercise prescription for all patients was similar to that reported previously for this population in the United States, Japan, and New Zealand [19,20,21], but the progression of exercise throughout cardiac rehabilitation allowed for a maximum contribution of approximately 30% to the exercise dose of 500 to 1000 MET·min per week recommended by the American Association of Cardiovascular and Pulmonary Rehabilitation, by the end of the rehabilitation program. This highlights the need for greater exercise progression within a cardiac rehabilitation program. Aerobic exercise intensity and dose along with resistance training load prescribed during 12 sessions of cardiac rehabilitation progressed more over the course of the program in some patient categories than others. Older patients experienced less frequent increases in their aerobic exercise prescription than their younger counterparts, while the aerobic exercise prescription provided to patients with the lowest initial physical capacity (less than 5 METs) did not progress. Moreover, the patients in the lowest tertile for initial grip strength had their resistance training prescription progressed less frequently than patients with higher initial grip strength. This limited progression in exercise prescription for the patients with the lowest initial exercise capacity and grip strength may be a function of the high relative exercise intensities that were prescribed to these patients at the beginning of the program.

Exercise progression (progressive overload) is a fundamental component of exercise prescription, to provide appropriate stimulus to derive physiological and muscular adaptations to increase physical capacity and reduce cardiovascular risk [10]. An increase in exercise dose above the individual’s accustomed amount of exercise, accomplished through increases in intensity, frequency, and/or duration, is required for overload to be achieved [22]. Increases in exercise frequency or duration have been reported to confer benefits in cardiac patients [23]; however, these variables did not change due to the design and availability of this program. It may not always be practical within the confines of structured sessions of a cardiac rehabilitation program to progress exercise frequency or duration, and therefore intensity is the prime candidate to provide a progressive overload, as occurred in the program that was the focus of this study. Further research is warranted to determine the best approach to providing greater exercise progression within cardiac rehabilitation, with consideration given to improved initial exercise prescription and progression, supplementing supervised exercise with an individually prescribed home-based program, the use of a telehealth approach to enable individuals to complete (or continue) their rehabilitation remotely to promote improved adherence, tailoring programs to look at outcomes rather than time for program completion, or the feasibility of offering a greater number of sessions if warranted based on symptom-limited exercise testing conducted at the end of the standard program.

Cardiac rehabilitation aims to improve physical capacity and strength to assist patients to resume their normal functioning, and as such, the exercise prescription should progress to an intensity that would allow patients to independently perform a wide range of activities of daily living within their individual capacity and goals. The attainment of this requires the exercise to be of an appropriate intensity and is likely to require more than 12 sessions for some patients. All patients within this study had an initial physical capacity greater than the MET value required for showering, getting dressed, vacuuming and hanging up washing. However, the progression of absolute aerobic exercise intensity for patients with the lowest initial physical capacity in this study was such that some of these patients did not achieve an intensity above that required to hang washing by the end of the program, and the mean absolute aerobic exercise intensity prescribed to patients with the highest initial physical capacity did not progress to that required for mowing the lawn, despite these patients having a final physical capacity that well-exceeded this level. This suggests that higher initial prescription and/or greater progression could have been prescribed for this latter group, a scenario that could be facilitated by more frequent assessment of aerobic capacity. Alternatively, if a conservatively ‘low’ intensity is prescribed at the commencement of the program, the desired levels may still be attainable upon completion, if the program is extended to include additional sessions. The inclusion of symptom-limited exercise testing at the conclusion of the exercise training program would assist practitioners in assessing the necessity of additional sessions on an individual basis. This might be preferred if there are concerns over the safety of higher intensity exercise: but would incur additional cost, with the additional attendance also being subjected to patient attendance barriers.

The weight lifted during resistance training did not progress to one requiring equivalent work to opening a car door for patients in any strength tertile. Additionally, the majority of patients with the lowest initial strength did not progress to an equivalent workload to that required for opening a refrigerator door or vacuuming during the program, with the mean value for this group remaining below this level. Conducting graded exercise tests and muscle strength tests as part of the routine assessment prior to a cardiac rehabilitation program, which is not currently specified in the Australian guidelines for cardiac rehabilitation [12], might provide more detailed information about patient capabilities upon which to base decisions about the level of strength that needs to be attained and enable the exercise dose to be more individually tailored. Furthermore, the identification of a minimum physical capacity based on patient goals for independent achievement of activities of daily living as an endpoint target for a cardiac rehabilitation program rather than conducting programs over a predefined number of sessions, and/or advocated transition to a maintenance or community-based program, may also provide greater health as well as physical capacity outcomes for the individual.

It has been suggested that programs limited to 12 to 18 sessions are not of sufficient duration for adequate progression of the exercise dose [22]. The mean cardiac rehabilitation program duration in Australia is 11.3 ± 5.5 sessions [4], which is reflected in the program evaluated within this study. It is noted that despite this relatively short duration, aerobic exercise dose and resistance training load were progressed. In part this may have been possible due to the low initial exercise prescription received by patients with moderate to high initial physical capacity or strength that allowed for greater progression for these patients in short periods of time. Again, if a conservatively low commencing dose is desired in the initial sessions this will enable progression, but it is possible that having started at a low level, more sessions may be needed for the progression to enable the patients to attain their full potential. Conversely, patients with the lowest initial physical capacity (less than five METs) or in the lowest tertile for initial grip strength, were prescribed the highest relative workloads and did not progress over the course of the cardiac rehabilitation program. Given that these patients are working ‘relatively harder’ than their ‘stronger’ compatriots, this suggests that the commencing exercise dose needs to be assessed, and a lower starting value could be considered, although some individuals might then require more sessions with ongoing progression in order for their target levels of strength to be attained. Conversely, it may be necessary to start the exercise prescription at a higher intensity to tailor the program for those with higher capacities. A concurrent solution could be the utilisation of baseline exercise testing data rather than patient symptoms as the basis for individualised initial exercise prescription, which might result in higher exercise intensities being prescribed initially to patients with higher physical capacity, and thereby allow for adequate exercise progression for maximising possible improvements in physical capacity and muscular strength for all patients during cardiac rehabilitation. Furthermore, it would seem appropriate for the dose of strength training within the sessions to need to exceed the level of strength required to perform basic activities of daily living according to individual goals—which in the current scenario may not be the case for all patients.

This study is limited by the incomplete data sets due to equipment (cycle ergometer) not sufficiently able to set desired workloads on some occasions. However, clear differences in the available data are demonstrated. The relatively small data-set though, means that findings related to age, cardiovascular intervention and physical capacity categories should be considered with a degree of caution. Despite these limitations, the data are reflective of published literature and, therefore, could be confidently referred to when developing cardiac rehabilitation programs. The low number of females (7%) who completed sufficient cardiac rehabilitation sessions to be included in this study means that the findings may not be truly representative of other Australian cardiac rehabilitation populations (other studies conducted in Australian cardiac rehabilitation populations have reported 20–30% female participants [24,25]). This is however reflective of adherence studies which have shown higher dropout and lower session attendance in females compared to males [26]. Exercise prescription practices are unlikely to be significantly different between males and females though.

## 5. Conclusions

Although aerobic exercise dose increased throughout cardiac rehabilitation, with exercise intensity being the component that was progressed, the final aerobic exercise dose achieved within cardiac rehabilitation by the patients in this study was below international recommendations for cardiac populations, and was prescribed at 64% of individual capacity. Patients with the lowest initial physical capacity (less than 5 METs) and those in the lowest tertile for initial grip strength were prescribed an exercise intensity that was a higher proportion of their initial functional capacity, and therefore received less frequent progression in their exercise prescription than those with higher capacity or grip strength. The overall findings of this study suggest that: (i) individual assessments of physical capacity would aid in the prescription of appropriate commencing exercise intensities and doses and should be recommended in guidelines; (ii) ongoing assessments would facilitate appropriate progression in exercise intensity and dose; (iii) completion of a program should be based on the patient attaining appropriate levels of functional capacity and/or reducing their cardiovascular risk to a minimum threshold. The inclusion of formal exercise testing as standard practice in Australian cardiac rehabilitation programs might better facilitate individualised exercise prescription and progression, leading to a higher exercise dose being completed, and subsequently greater improvements in physical capacity and muscular strength, cardiovascular risk profile and quality of life.

## Figures and Tables

**Table 1:** Baseline demographic information for included participants.

Variable	Outcome
Sex	
Male	56
Female	4
Age (mean ± SD)	61.8 ± 10.8 years
<65 years	35
≥65 years	22
BMI (mean ± SD)	29 ± 5 kg/m^−2^
Initial physical capacity (mean ± SD)	6.1 ± 1.3 METs
<5 METs	9
5–7 METs	32
>7 METs	19
Cardiovascular intervention	
Surgical	22
Non-surgical	35
Comorbidities	
Type 2 diabetes mellitus	6
Orthopaedic conditions	16
Angina	9
Medication use	
Lipid lowering only	13
BP lowering only	6
Lipid and BP lowering	38

BP, blood pressure; METs, metabolic equivalents; SD, standard deviation.

## Data Availability

The data is securely stored in the RMIT University archives in accordance with University policy. Data is available on request to the corresponding author, and is subject to privacy and confidentiality, in accordance with the ethics approval for the project.
